# Assembly Factor Omp85 Recognizes Its Outer Membrane Protein Substrates by a Species-Specific C-Terminal Motif 

**DOI:** 10.1371/journal.pbio.0040377

**Published:** 2006-11-07

**Authors:** Viviane Robert, Elena B Volokhina, Freya Senf, Martine P Bos, Patrick Van Gelder, Jan Tommassen

**Affiliations:** 1 Department of Molecular Microbiology and Institute of Biomembranes, Utrecht University, Utrecht, The Netherlands; 2 Department of Molecular and Cellular Interactions, Flanders Interuniversity Institute for Biotechnology (VIB), Free University Brussels, Brussels, Belgium; Tufts University School of Medicine, United States of America

## Abstract

Integral β-barrel proteins are found in the outer membranes of Gram-negative bacteria, mitochondria, and chloroplasts. The assembly of these proteins requires a proteinaceous apparatus of which Omp85 is an evolutionary conserved central component. To study its molecular mechanism, we have produced Omp85 from Escherichia coli in inclusion bodies and refolded it in vitro. The interaction of Omp85 with its substrate proteins was studied in lipid-bilayer experiments, where it formed channels. The properties of these channels were affected upon addition of unfolded outer-membrane proteins (OMPs) or synthetic peptides corresponding to their C-terminal signature sequences. The interaction exhibited species specificity, explaining the inefficient assembly of OMPs from *Neisseria* in E. coli. Accordingly, the in vivo assembly of the neisserial porin PorA into the E. coli outer membrane was accomplished after adapting its signature sequence. These results demonstrate that the Omp85 assembly machinery recognizes OMPs by virtue of their C-terminal signature sequence.

## Abstract 2

OMP85 is an evolutionarily conserved protein necessary for the assembly of integral beta-barrel proteins in bacteria. The authors show that OMP85 forms a channel and recognizes its unfolded subtrates through a C-terminal signature sequence.

## Introduction

The cell envelope of Gram-negative bacteria consists of two membranes, the inner membrane (IM) and the outer membrane (OM), which are separated by the peptidoglycan-containing periplasm. In contrast to integral IM proteins, which span the IM by hydrophobic α-helical segments, integral OM proteins (OMPs) present an entirely different structure, the β-barrel. They consist mainly of antiparallel amphipathic β-strands, exposing the hydrophobic residues to the lipid environment of the membrane and the hydrophilic ones to the interior of the barrel [1]. Similar β-barrel proteins are found in the OMs of eukaryotic cell organelles of endosymbiont origin, mitochondria and chloroplasts.

OMPs are synthesized in the cytoplasm as precursors with an N-terminal signal sequence, which is essential for translocation across the IM via a translocation apparatus that is composed of the Sec proteins. Extensive genetic and biochemical analyses have unraveled many molecular details of the process of protein translocation across the IM [2]. The later stages in OMP biogenesis, i.e., transport through the periplasm and assembly into the OM, are much less well understood. It has been demonstrated that the acquisition of tertiary structure precedes, at least partially, the insertion of the proteins into the OM [3]. Several periplasmic proteins have been shown to play a role in OMP biogenesis. One of them, the chaperone SurA, stimulates OMP folding [4]. Another one, designated Skp, binds OMPs directly after they emerge from the Sec channel [5]. It may play a role in the targeting of OMPs to their assembly sites [5,6] or prevent premature folding or aggregation [7].

Recent studies have led to the identification of a proteinaceous apparatus that is dedicated to the insertion and assembly of OMPs. An OMP of *Neisseria meningitidis,* designated Omp85, was shown to be essential for the viability of the bacteria, and its depletion in a genetically engineered strain resulted in the periplasmic accumulation of unassembled forms of all OMPs tested [8]. Omp85 homologs were found in all Gram-negatives studied [9], and the Escherichia coli homolog, which is encoded by the *yaeT* gene, was demonstrated to be essential and required for OMP biogenesis as well [10–12]. Semi-native SDS-PAGE revealed that N. meningitidis Omp85 is part of a high molecular weight oligomeric complex of unknown composition [8]. In the case of *E. coli,* Omp85 was shown to be part of a hetero-oligomeric complex with three lipoproteins, YfgL, NlpB, and YfiO [12]. YfgL and YfiO appear to play a role in OMP biogenesis as well, since the corresponding mutants showed decreased OMP levels [12,13]. Interestingly, a mitochondrial Omp85 homolog, called Omp85, Tob55, or Sam50, has been identified and shown to be essential for the assembly of β-barrel proteins into the OM of these organelles, demonstrating the evolutionary conservation of OMP assembly [14–16]. Another Omp85 homolog, designated Toc75, is a central component of the protein import machinery in the chloroplast OM [17] and may be directly involved in OMP assembly as well [18], although this membrane contains additional Omp85 homologs that may fulfill this function [19]. Also the bacterial outer membrane contains additional, distantly related Omp85 homologs, generically designated TpsB, which have a different function, i.e., they are the key components in the Two-Partner Secretion (TPS) pathway [20]. Apart from the *yaeT* gene, the E. coli K-12 genome additionally contains a distant *omp85* homolog, *ytfM*. Homologous genes, closely related to *ytfM,* are widely disseminated among Gram-negatives, but their function has not yet been experimentally addressed.

After the identification of its components, the structure and functional mechanism of the OMP assembly machinery await further analysis. We have presented a topology model for Omp85, consisting of a periplasmic N-terminal domain and a C-terminal OM-embedded β-barrel domain [8]. Furthermore, we have postulated that the Omp85 complex might form a pore that can laterally open to allow for the insertion of nascent OMPs into the OM [21]. Another key question is how the machinery selects its substrates. Evidence for a direct interaction between the neisserial Omp85 complex and its substrates was obtained in Far-Western blot analysis, which demonstrated binding of non-native PorA, one of the major porins of *N. meningitidis,* to the oligomeric Omp85 complex immobilized on a nitrocellulose membrane [8]. We hypothesized [9,21] that the N-terminal periplasmic domain of Omp85 might recognize the signature sequence that was previously detected at the C-termini of the vast majority of bacterial OMPs [22]. This signature sequence consists of a Phe (or Trp) at the C-terminal position, and hydrophobic residues at positions 3 (preferentially Tyr), 5, 7, and 9 from the C-terminus. Deletion or substitution of the C-terminal Phe drastically affected the assembly efficiency of the OM porin PhoE in E. coli [22].

In the present work, we have purified Omp85 from E. coli to test the various hypotheses outlined above. We demonstrate that Omp85 forms oligomers and exhibits pore activity upon reconstitution in lipid bilayers. We also present evidence that the C-terminal OMP signature sequence is the sorting signal recognized by Omp85 and that the previously observed conditional lethality of the expression of neisserial OMPs in E. coli [23] is due to divergence of this signature sequence between the two organisms.

## Results

### E. coli Omp85 Can Be Refolded from Inclusion Bodies

**Figure 1:**
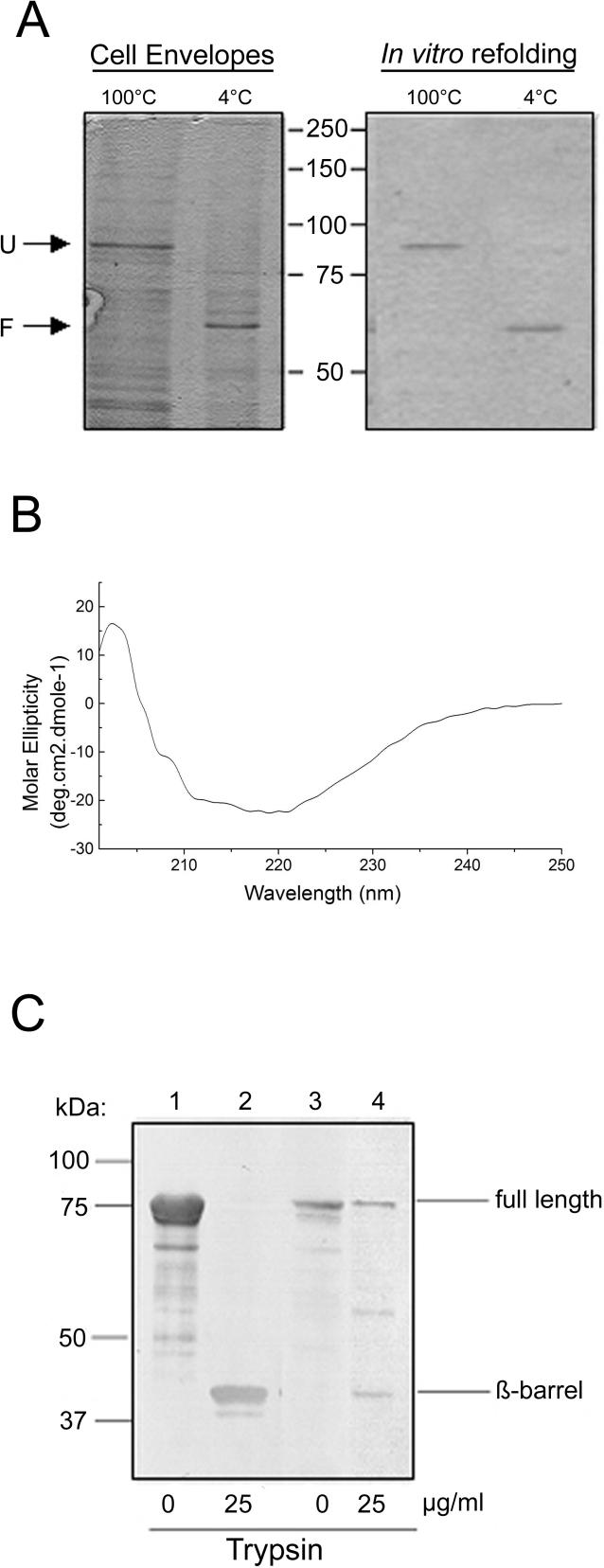
E. coli Omp85 Exhibits a β-Barrel Structure (A) Comparison of the heat modifiability of in vitro folded Omp85 (right panel) and Omp85 from cell envelopes of E. coli strain AM1095 pCLyaeT (left panel) in SDS-PAGE. Samples were either heated to 100 °C or not before loading on the gel as indicated above the lanes. Only the relevant part of the gels is shown and the positions of molecular mass marker proteins are shown in the center (in KDa). F, folded Omp85; U, unfolded Omp85. (B) CD spectrum of Omp85. (C) Immunoblot showing tryptic fragments of refolded Omp85 (lanes 1 and 2) and TOP10F′ cell envelopes (lanes 3 and 4). The blot was probed with anti-Omp85 antiserum. The fragment indicated as β-barrel was N-terminally sequenced.

The E. coli Omp85 was overproduced without its signal sequence resulting in the formation of cytoplasmic Omp85 aggregates (inclusion bodies). After solubilization of the inclusion bodies in 8 M urea, the protein was refolded in vitro by dilution in a buffer containing the detergent 3-dimethyldodecylammoniopropane-sulfonate (SB12). Folding was monitored by semi-native SDS-PAGE making use of the heat-modifiable character of bacterial OMPs, i.e., folded monomers generically migrate faster in SDS-PAGE gels than the heat-denatured proteins [24]. Accordingly, SDS-PAGE analysis of cell envelopes of E. coli overexpressing wild-type Omp85 from a low copy number vector showed that the protein migrated at approximately its predicted molecular weight (90 KDa) after denaturation of the sample, but considerably faster when the sample was not boiled (Figure 1A, left panel). Of note, no oligomeric forms with a lower electrophoretic mobility were detected under these conditions, neither on the gel nor on Western blots (unpublished data). Since the in vitro folded Omp85 displayed similar heat modifiability as Omp85 in cell envelopes (Figure 1A), we concluded that the refolded protein had acquired its native conformation. Moreover, the circular dichroism spectrum of the purified refolded protein revealed a high content of β-strand, representative of OMP secondary structure (Figure 1B). We have proposed a topology model for Omp85, consisting of a periplasmic N-terminal domain and a C-terminal OM-embedded β-barrel [8]. To probe this two-domain model and to further assess the correct folding of the protein, cell envelopes and in vitro folded Omp85 were treated with trypsin. Both preparations yielded a tryptic fragment of similar size (Figure 1C), substantiating the correct conformation of the refolded protein. Generically, the β-barrel domains of OMPs are highly resistant to proteases. Indeed, the major tryptic fragment from the refolded protein represents an intact β-barrel as indicated by its N-terminal sequence, which was found to be NTGSF, a sequence located just upstream of the proposed β-barrel domain, and by its β-strand composition, as confirmed by circular dichroism measurements (unpublished data).

### Omp85 Forms Oligomeric Structures

**Figure 2:**
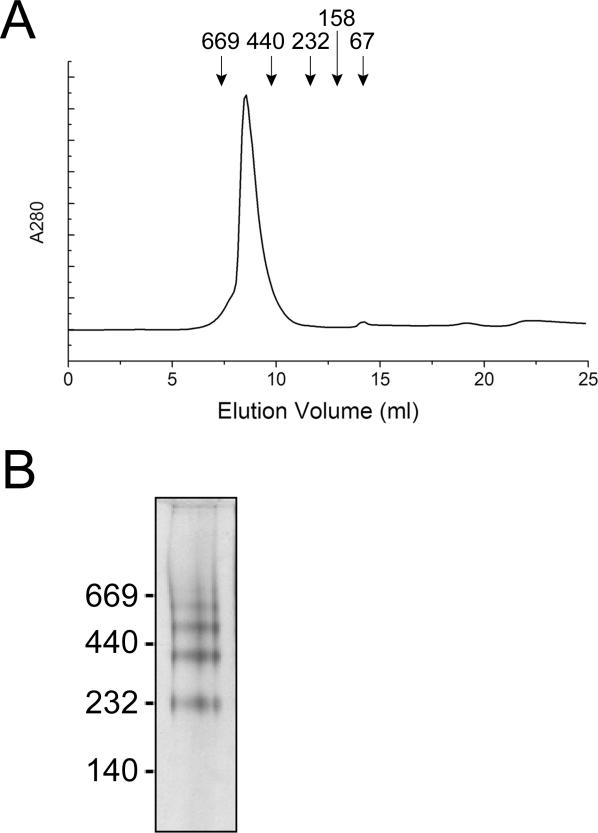
E. coli Omp85 Forms Multimers (A) Size exclusion chromatography of purified refolded Omp85. Symbols indicate elution volumes of the MW standard proteins thyroglobulin (669 KDa), ferritine (440 KDa), catalase (232 KDa), aldolase (158 KDa), and ovalbumin (67 KDa) (Amersham Bioscience). (B) Blue Native PAGE gel of purified Omp85. The positions of molecular mass marker proteins are shown at the left (in KDa).

Some members of the Omp85 superfamily were reported to form oligomers [16,25]. The capacity of E. coli Omp85 to form oligomers was assessed by size-exclusion chromatography. As shown in Figure 2A, Omp85 eluted with a retention time consistent with an apparent molecular mass of approximately 473 KDa, indicating that Omp85 indeed forms oligomers, probably tetramers. However, the exact oligomeric configuration is difficult to assess in this assay, since the contribution of the detergent micelle to the apparent molecular mass of the complex is not exactly known. Blue Native PAGE confirmed the formation of Omp85 oligomers (Figure 2B). The four distinct bands detected likely represent mono-, di-, tri-, and tetramers, but the uncertainty of the contribution of the Coomassie blue dye to the electrophoretic mobility precludes a definitive determination. The detection of multiple bands in the Blue Native PAGE, in contrast to the symmetrical peak in the gel filtration assay, can be explained by assuming that the oligomer is in equilibrium with smaller oligomers and monomers, in which case binding of Coomassie to the subunit interface is expected to shift the equilibrium.

### Omp85 Forms Channels

**Figure 3:**
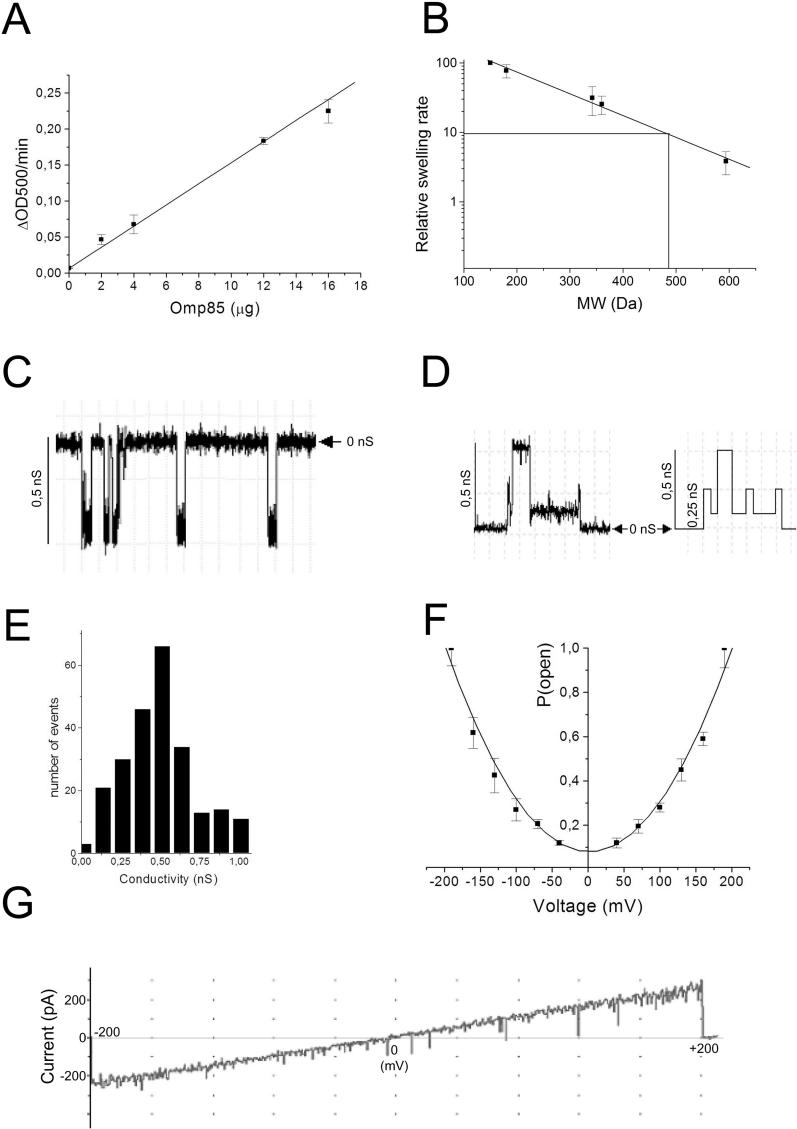
Pore-Forming Activity of Omp85 (A) Swelling rates of proteoliposomes reconstituted with the indicated amount of Omp85 in iso-osmotic solutions of L-arabinose. (B) Relative swelling rates of proteoliposomes containing Omp85 in solutions of sugars with different MW. The sugars used were arabinose (150 Da), glucose (180 Da), saccharose (342 Da), maltose (360 Da), and raffinose (594 Da). The data are shown relative to the swelling in arabinose and are the averages of at least four independent experiments. The indicated swelling rate corresponding to 10% of that in arabinose served to estimate the size of the channel. (C) Recording of Omp85 pores formed in planar lipid bilayers at an applied potential of −150 mV. (D) Current recordings showing sub-conductance states of Omp85 channels and its idealized current trace (right panel). The horizontal arrowhead shows the zero-conductance level. (E) Amplitude histogram of current derived from channel openings at +50, +100, and +150 mV. Results from four experiments were pooled. (F) Voltage dependence of the probability for the Omp85 channel of being in its open state. Data were normalized relative to the maximal mean current at 190 mV. Data points represent averages of two independent bilayers. (G) Voltage-ramp analysis of Omp85 channels from 0 to 200 mV and from 0 to −200 mV. In the experiment shown, eight 0.12-nS channels were present in the bilayer.

We have suggested that Omp85 may form pores, used for the insertion of OMPs. Pore formation by Omp85 was determined in liposome-swelling assays and planar lipid bilayers (Figure 3). In liposome-swelling assays, pore-forming activity of Omp85 was clearly detected by the swelling of the proteoliposomes suspended in an iso-osmotic arabinose solution. The permeability of liposomes toward arabinose was linearly proportional to the amount of Omp85 added (Figure 3A). No swelling was noticed in liposomes without proteins (unpublished data). The specific activity of Omp85-containing proteoliposomes toward arabinose was approximately 0.019 ΔOD_500_ (decrease in optical density) per min/μg protein, which is much less than reported for the E. coli porins OmpF or OmpC [26], but equivalent to that of HMW1B protein, an Omp85-like protein belonging to TpsB family [25]. The size of the Omp85 channel was estimated by plotting the diffusion rates of solutes of different sizes (Figure 3B). The molecular weight of a solute that results in 10% of the swelling activity of arabinose can be used to calculate the diameter of the channels [27]. The calculated channel size of the Omp85 pores was again rather similar to that of HMW1B [25], i.e., approximately 2.5 nm in diameter. This large channel size on the one hand, but the low specific activity of the proteoliposomes on the other hand suggest that only a fraction of the Omp85 protein population might be in an open conformation.

To study the pore characteristics of Omp85 in further detail, planar lipid bilayer experiments were performed. Conductance measurements revealed the opening and closing of pores (Figure 3C). The majority of the conductance steps was of approximately 0.5 nS in 1 M KCl (Figure 3C and 3E), but subconductance states of 0.12 nS were seen when the majority of the channels was in a non-active state (Figure 3D). This observation suggests that the different amplitude levels correspond to a tetramer of Omp85, in which every subunit forms a pore that can open and close independently. Because of the low conductivity observed, the size of the channel was not estimated by this technique.

Voltage-ramp recordings of multiple channels (Figure 3G) revealed a linear current/voltage relationship, showing that the conductance of the pores was constant over the entire voltage range tested and identical at positive and negative potentials.

The probability of the Omp85 channels to be open at different membrane potentials was determined after allowing the channels to adapt to various membrane potentials during 2 min. The currents subsequently measured showed an exponential relation to the magnitude of the applied membrane potential, indicating that these channels are voltage activated (Figure 3F).

### OMPs Can Modulate Omp85 Channel Properties

**Figure 4:**
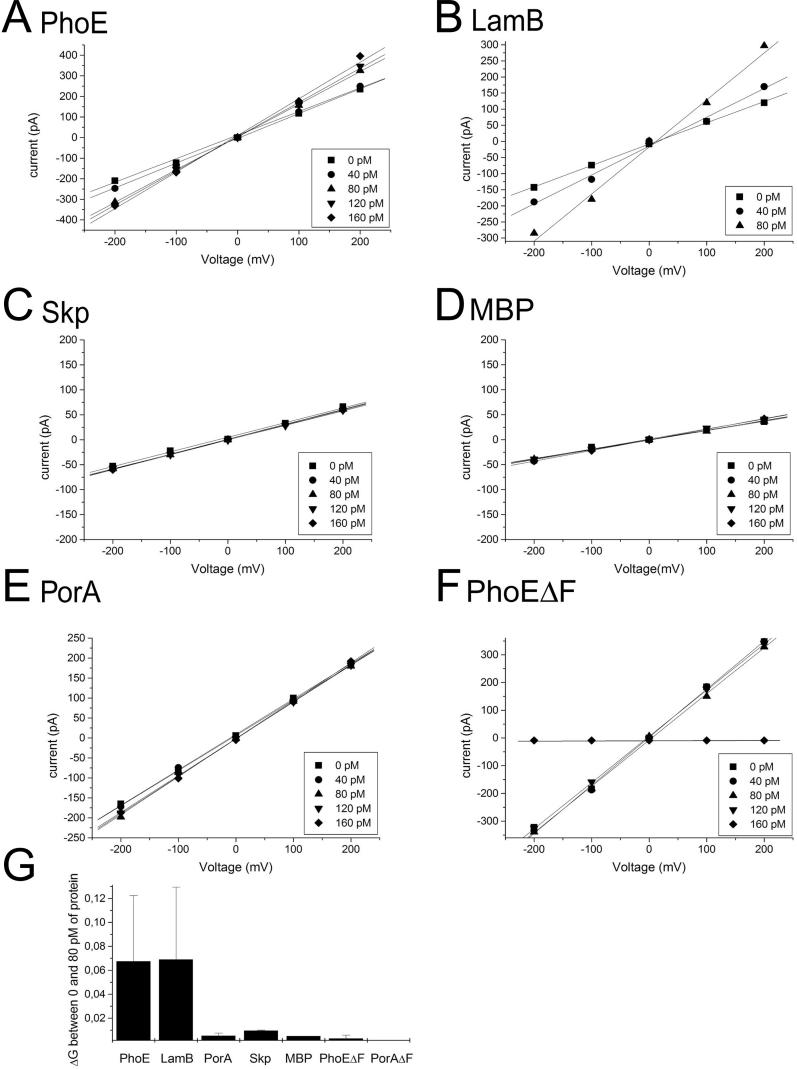
Denatured OMPs Affect Omp85 Pore Activity (A–F) Voltage ramps were applied to bilayers containing several Omp85 channels before and after the addition of the proteins indicated above the panels. The amounts of protein added are shown in the inserts. The numbers of active 0.12-nS Omp85 channels in the experiments depicted were 10, 5, 3, 2, 7, and 13, in (A), (B), (C), (D), (E), and (F), respectively. (G) Increase in conductivity per Omp85 channel upon addition of 80 pM of each protein. Each experiment was repeated at least four times.

We have previously shown in Far-Western blotting experiments that Omp85 from N. meningitidis interacts with the non-native N. meningitidis porin PorA [8]. We investigated whether interaction with substrates affects the channel activity of Omp85 reconstituted in a planar lipid bilayer (Figure 4). Denatured E. coli PhoE porin was added to both the *cis* and the *trans* compartment, since Omp85 probably assembles in the bilayers in random orientation. The addition of pM concentrations of PhoE induced a dose-dependent opening of the Omp85 channels (Figure 4A) indicating that PhoE protein bound to the reconstituted Omp85 and affected its channel characteristics. Similarly, the addition of another OMP from *E. coli,* the maltoporin LamB, caused the Omp85 channels to open (Figure 4B), whereas two periplasmic proteins, the chaperone Skp and the maltose-binding protein (MBP), did not affect the channels (Figure 4C and 4D). The addition of 80 pM of denatured PhoE or denatured LamB increased the conductivity per 0.12-nS Omp85 channel by approximately 0.067 and approximately 0.069 nS, respectively, corresponding to an increase of approximately 56%–58% (Figure 4G).

### Identification of the Recognition Signal for Omp85 in Its Substrate Proteins

The vast majority of bacterial OMPs contain a C-terminal signature sequence, of which a Phe (or Trp) at the ultimate C-terminal position is the most prominent feature [22]. We reasoned that this signature sequence might represent a sorting signal that is recognized by Omp85. To test this possibility, we investigated whether a mutant PhoE protein that lacks the C-terminal Phe residue (PhoEΔF330) affects the Omp85-channel properties. Remarkably, in contrast to wild-type PhoE, the addition of denatured PhoEΔF330 to the bilayers did not open the channels, but, at higher concentrations, even completely blocked the channels (Figure 4F). Apparently, the mutant protein affects the Omp85 channels differently. Blockage of the Omp85 channels by the mutant PhoE appeared irreversible, since the subsequent addition of wild-type PhoE did not activate the channels (unpublished data).

**Figure 5:**
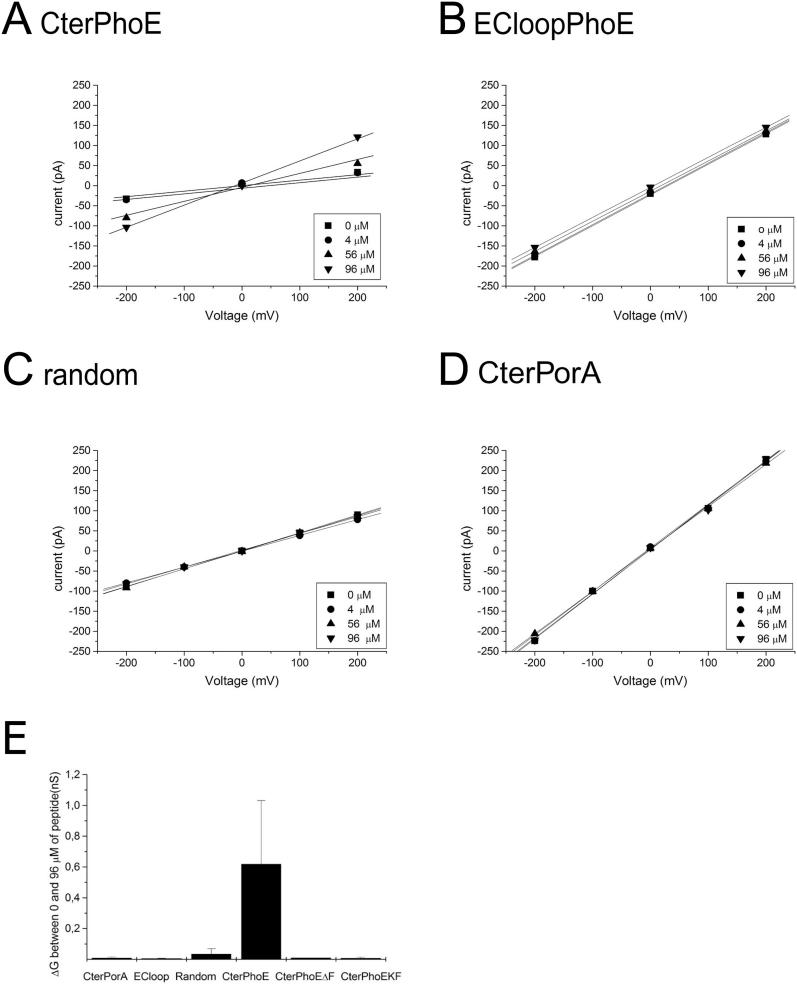
C-Terminal PhoE Peptide Affects Omp85 Pore Activity (A–D) Voltage ramps were applied to bilayers containing Omp85 channels before and after the addition of the peptides shown above the panels. The amounts of peptides added are shown in the inserts. The numbers of active 0.12-nS Omp85 channels in the experiments depicted were 1, 6, 4, and 9, in (A), (B), (C), and (D), respectively. (E) Increase in conductivity per Omp85 channel upon addition of 96 μM of each peptide. Each experiment was repeated at least four times.

Next, we investigated whether the C-terminal OMP signature is sufficient for recognition by Omp85 (Figure 5). A synthetic peptide corresponding to the C-terminal 12 amino acid residues of PhoE was added to planar lipid bilayers containing Omp85 channels. At μM concentrations, the peptide induced a dose-dependent opening of the Omp85 channels (Figure 5A). Essentially, the addition of 96 μM of the peptide increased the conductivity per 0.12-nS Omp85 channel by on average approximately 0.6 nS (Figure 5E). A similar 11-residues peptide lacking the C-terminal Phe (CterPhoEΔF) did not affect the Omp85 channel properties at the same (Figure 5E) and even higher concentrations (tested up to 136 μM) (unpublished data), showing that, in the peptide also, the C-terminal Phe is important for the proper interaction with Omp85. As further controls, we used a peptide corresponding to ten amino acid residues from the first extracellular loop of PhoE and a peptide containing the last 12 amino acids of PhoE, but in a randomized order. Neither peptide affected the conductivity of the Omp85 channels (Figure 5B and 5C).

### The Omp85–Substrate Interactions Are Species Specific

It has been reported repeatedly that the expression of neisserial OMPs in E. coli causes lethality problems [23,28,29]. We hypothesized that these problems may be due to no or inadequate recognition of the neisserial OMPs by E. coli Omp85. To test this possibility, we investigated whether the porin PorA from N. meningitidis affected the E. coli Omp85 channels in the planar lipid bilayers. Indeed, neither PorA (Figure 4E) nor its C-terminal peptide (Figure 5D) was able to activate the Omp85 channels, whereas a mutant form of PorA lacking the C-terminal Phe (PorAΔF) did not block them (Figure 4G).

The failure of PorA or its C-terminal peptide to affect E. coli Omp85 channels in the planar lipid bilayer assay was surprising, since PorA does contain Phe at the ultimate position. Also in other respects, the C-terminal end of PorA (AASVGLRHKF) does comply to the consensus of the C-terminal OMP signature sequence, with hydrophobic residues being present at positions 5 (Leu), 7 (Val), and 9 (Ala) from the C-terminus. Only at position 3 from the C-terminus, PorA contains His, rather than Tyr or a hydrophobic residue as present in the consensus sequence. To determine whether this is a general feature of neisserial OMPs, the C-terminal sequences of representative OMPs were compared (Table 1). No particular characteristics of the amino acid at position 3 from the C-termini were evident. However, it was remarkable that the vast majority of neisserial OMPs contain a positively charged residue (Arg or Lys) at the penultimate position. With a few exceptions (e.g., the siderophore receptor FhuA), this is not the case in enterobacterial OMPs. Therefore, the residue in the penultimate position could be involved in the species specificity of Omp85 recognition.

**Table 1:**
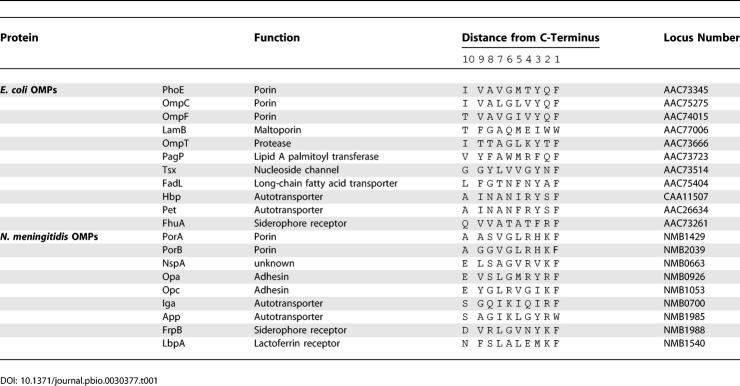
C-Terminal Ten Amino Acids of Selected E. coli and *N. meningitidis* OMPs

**Figure 6:**
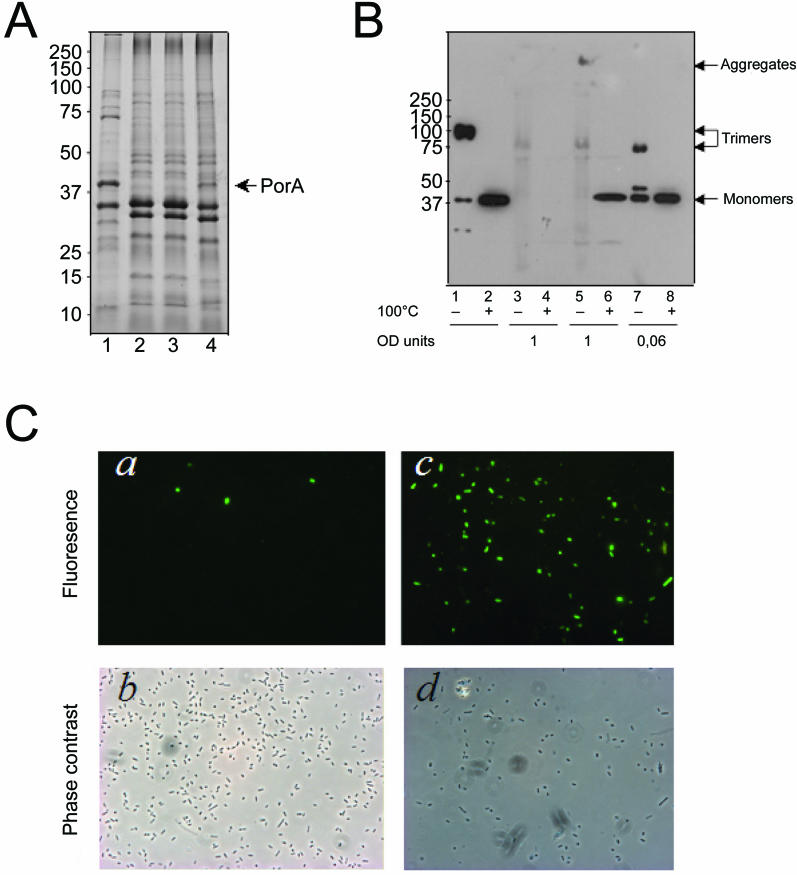
The Species Specificity of OMP Insertion between N. meningitidis and E. coli Is Related to the Nature of the C-Terminal Signature Sequence (A) Coomassie-stained SDS-PAGE gel showing cell envelopes from N. meningitidis strain HB-1 (lane 1), and E. coli containing pCRII-TOPO (lane 2), pII-*porAwt* (lane 3), or pII-*porA-Q* (lane 4). (B) Cell envelopes were analyzed by SDS-PAGE after denaturation (+) or without denaturation (–) at 100 °C as indicated beneath the lanes. The gel was blotted and probed with anti-PorA antibody MN5C11G. Cell envelopes were from N. meningitidis strain HB-1 (lanes 1 and 2), and from E. coli carrying either pCRII-TOPO (lanes 3 and 4), pII-*porAwt* (lanes 5 and 6), or pII-*porA-Q* (lanes 7 and 8). Note the different loadings, which are indicated in optical density (OD) units of the original cultures below the blot. In (A) and (B), the E. coli strains were grown with 0.5 mM IPTG, and the positions of molecular mass marker proteins are shown at the left (in KDa). (C) Immunofluorescence microscopy analysis. The binding of a PorA-specific mAb to E. coli Top10F′ cells expressing wild-type PorA *(a)* or mutant PorA-Q *(c)* was evaluated. The corresponding phase-contrast images are shown in *(b)* and *(d)*, respectively.

To determine whether the species specificity of Omp85 recognition is indeed related to the nature of the amino acid residue at the penultimate position, a synthetic peptide (CterPhoEKF) corresponding to the C-terminal 12 amino acid residues of PhoE but with Lys instead of Gln at the penultimate position was added to planar lipid bilayers containing Omp85 channels. In contrast to the corresponding wild-type peptide, this peptide did not activate the Omp85 channels (Figure 5E). Additionally, we tested the role of the penultimate amino acid in vivo. The Lys present in this position in PorA was substituted by Gln, which is found in this position in PhoE and many other E. coli OMPs. The wild-type and the mutant *porA* allele, designated *porA-Q,* were cloned behind an IPTG-inducible promoter and introduced into E. coli. In contrast to the wild-type PorA, the PorA-Q mutant protein was detected in the cell envelope protein patterns after IPTG induction (Figure 6A). After overloading of the gels, however, also the wild-type PorA could be detected on Western blots (Figure 6B, lane 6). When the samples were left unboiled before electrophoresis, a considerable proportion of PorA-Q produced in E. coli migrated in the gel as trimers, indicating that the protein was correctly assembled in the heterologous host (Figure 6B, lane 7). These trimers had a higher electrophoretic mobility than the PorA trimers produced in the authentic host (Figure 6B, lane 1), since in *Neisseria* they are firmly associated to the RmpM protein, which stabilizes them [30]. When membranes from E. coli expressing the wild-type PorA were analyzed, no PorA trimers were detectable (Figure 6B, lane 5; note that the faint band in lane 5, migrating slightly slower than the PorA-Q trimers in lane 7, is unspecific since it is also visible in lane 3 containing control cells not expressing any PorA). The only PorA-specific signal in the unboiled sample was found at the position of the stacking gel, indicative of PorA aggregates. The total signal in the native sample was lower than in the denatured sample (lane 6), presumably because the aggregates do not blot very efficiently.

Correct assembly of PorA into the E. coli OM was also assessed by investigating its cell surface exposure in immunofluorescence microscopy with a monoclonal antibody (mAb) directed against the cell surface–exposed loop 4 of PorA (Figure 6C). Whereas the cells expressing wild-type PorA were barely labeled, the majority of the cells expressing the mutant protein PorA-Q were densely labeled over the entire cell surface. Collectively, these data demonstrate that wild-type PorA expressed in E. coli is poorly assembled and prone to aggregation and, presumably, degradation, and that the amount and assembly of PorA in E. coli can be vastly increased by adjusting the penultimate amino acid.

## Discussion

Omp85 is an essential component required for the assembly of OMPs. It is present in all Gram-negative bacteria and even in eukaryotic cell organelles of endosymbiont origin, where it performs a similar function. So far, knowledge of the structure and functional mechanism of the protein is extremely scarce. We have shown previously that Omp85 of N. meningitidis is part of a high molecular weight complex that binds non-native porins [8]. Since E. coli Omp85 is part of a complex containing several additional components [12], it was not clear which subunit interacts with the substrate OMPs. To facilitate structural and mechanistic studies, we have produced Omp85 from E. coli in inclusion bodies and refolded it in vitro into its native conformation. This procedure yields large quantities of protein, which may be suitable for structural studies by X-ray crystallography, as was successfully demonstrated previously for several other OMPs (e.g., see [31]). The refolded Omp85 formed oligomers, possibly tetramers, as evidenced by size exclusion chromatography and Blue Native PAGE.

Omp85 showed a clear channel activity when incorporated in liposomes or planar lipid bilayers. The liposome swelling assay suggested the presence of fairly large channels with a diameter of approximately 2.5 nm, comparable to the channels formed by the TpsB protein HMW1B of *Haemophilus influenzae,* a distantly related member of the Omp85 superfamily [25]. This diameter seems large enough to tolerate insertion of denatured or intermediate folded OMPs. The specific activity of the Omp85 channels for solute diffusion, however, appeared very low, suggesting that only a fraction of the protein population might be in an open conformation. Consistently, planar lipid bilayers experiments revealed an increased frequency of channel openings upon application of a transmembrane potential. Similar voltage activation has been reported for several other OM channel proteins, including, for example, the secretin PulD, which is involved in type II protein secretion [32]. The physiological significance is unclear; it probably does not reflect the energy status of the cell, since the OM is not energized by the presence of a respiratory chain generating a proton-motive force. Additionally, the Donnan potentials across the OM are generally rather low [33]. Hence, the voltage gating observed in vitro might be unrelated to the in vivo functioning of the protein.

The majority of the channel openings and closings observed in the planar lipid bilayers corresponded to channels with a conductance of approximately 0.5 nS. These pores are similar in size to the 410-pS pores formed by SynToc75, a cyanobacterial homolog of the chloroplast protein-import component Toc75 and a distant member of the Omp85 superfamily [34]. It is conceivable that SynToc75 is the functional cyanobacterial homolog of Omp85. In the case of the Omp85 channels, subconductance states of approximately 0.12 nS were observed, suggesting that the 0.5 nS channels correspond to tetrameric Omp85 complexes, with each subunit forming an independent channel. Similarly, subconductance states of SynToc75 were reported [34] and HMW1B was reported to form tetramers [25].

Although the pore properties of Omp85 in vivo might be different from those measured in the planar lipid bilayers, e.g., because of the different lipid composition of the OM, the in vitro system provided a convenient assay to study the interaction of Omp85 with its substrate proteins. We established that Omp85 directly and specifically interacts with denatured OMPs causing opening of the channels, whereas periplasmic proteins had no effect on Omp85 pore activity. We considered the possibility that Omp85 recognizes its substrates by virtue of their C-terminal OMP signature sequence, which is found in the vast majority of bacterial OMPs. In vivo, it has been shown that the deletion or substitution of the C-terminal Phe, which is the most prominent feature of this signature sequence, in porin PhoE drastically affected the assembly of the protein into the membrane and was, at high expression levels, lethal to the cells [22]. Indeed, we found that the C-terminal Phe residue plays an important role in the recognition by Omp85, since a mutant PhoE lacking this residue, PhoEΔF330, did not open, but, at high concentrations, even completely blocked the Omp85 channel. Moreover, a synthetic peptide corresponding to the C-terminal 12 amino acid residues of PhoE also caused Omp85-channel opening, like the complete PhoE protein did. Hence, we conclude that the signature sequence is important for the appropriate interaction of nascent OMPs with the assembly machinery. Interestingly, the same OMP signature is recognized by the IM-bound protease DegS, thereby initiating a proteolytic cascade, eventually resulting in a periplasmic stress response when unfolded OMPs accumulate in the periplasm [35]. It is also interesting to note that another protease that participates in this signaling cascade, RseP (formerly designated YaeL), is encoded by a gene, which is located in most Gram-negative bacterial genomes directly upstream of the *omp85* gene.

Interestingly, the porin PorA from N. meningitidis did not stimulate the channel activity of E. coli Omp85 even though the protein has the C-terminal signature sequence. Similarly, a synthetic peptide corresponding to the C-terminus of PorA did not affect the Omp85 channels, suggesting divergence of the OMP signatures between the two species. Consistently, the expression of neisserial OMPs, like that of the mutant PhoE lacking the C-terminal Phe, has repeatedly been reported to be lethal in E. coli [23,29]. Comparison of the C-terminal sequences of neisserial and E. coli OMPs revealed a strong preference for a positively charged residue (Arg or Lys) at position 2 from the C-terminus in the neisserial OMPs, which could be responsible for the species specificity observed. Indeed, a synthetic C-terminal PhoE peptide with a Gln to Lys substitution at this position failed to activate the Omp85 channels in vitro, whereas a Lys to Gln substitution at this position in the meningococcal PorA drastically improved the assembly of the protein into the E. coli OM in vivo. Thus, even though the process of OMP assembly by an Omp85-related machinery is evolutionary conserved, species-specific adaptations appear to have occurred.

It should be noticed that the C-terminal Phe is not absolutely essential for recognition by Omp85. Whereas wild-type PhoE activated Omp85 channel activity, the PhoE mutant lacking the C-terminal Phe blocked the Omp85 pores, showing that it interacts with Omp85, albeit differently and less efficiently, since much higher concentrations of the mutant protein were required to affect Omp85 pore activity. Furthermore, whereas high-level expression of this mutant protein in vivo is lethal and leads to its periplasmic aggregation [22,36], low-level expression is tolerated and leads to the efficient assembly of the mutant protein into the OM [36]. Similarly, studies reporting the successful assembly of neisserial OMPs in the E. coli OM [37] may be explained by lower expression levels. These observations indicate that there are alternative, less-efficient recognition sites in the mutant PhoE protein and the neisserial OMPs that mediate binding to Omp85. An alternative explanation is that the C-terminal recognition site is not completely inactivated by the deletion of the phenylalanine or the presence of a positive charge at the penultimate position. However, the latter explanation seems less likely, since, in contrast to a synthetic peptide corresponding to the last 12 amino acid residues of PhoE, similar peptides lacking the C-terminal Phe or containing a positively charged residue at the penultimate position did not affect Omp85 channel properties at all. Kinetic partitioning between OM incorporation and aggregation of periplasmic OMP intermediates may explain the observation that OMPs with a defective C-terminal recognition sequence are assembled into the OM at low expression levels [36]. The presence of an appropriate recognition sequence at the C-terminus will assure efficient assembly into the OM even at high expression levels. In the absence of such a recognition sequence, secondary recognition sites may still mediate binding to Omp85, but the kinetics of incorporation into the OM will be reduced and the aggregation pathway will dominate. However, at lower expression levels, the kinetics of aggregation will be reduced, allowing the less-efficient OM incorporation pathway to become dominant again.

Which sequences in OMPs may function as secondary recognition sites for Omp85 interaction? The last ten amino acid residues of PhoE form an amphipathic β-strand. In the absence of the C-terminal Phe, Omp85 may recognize, though less efficiently, internal β-strands, many of which also end with an aromatic residue [38]. In some classes of OMPs, the C-terminal OMP signature could not be discerned [22]. Possibly, in these cases also, Omp85 recognizes an internal β-strand. Actually, one of the major OMPs of *E. coli,* OmpA, consists of two domains, an N-terminal β-barrel domain embedded in the OM and a C-terminal periplasmic domain. The OMP signature sequence was found in this case at the end of the β-barrel domain, i.e., internal in the primary structure of the protein [22], and deletion analysis showed that this sequence is required for binding of OmpA to the OM [39]. These data indicate that Omp85 may indeed recognize internal binding sites in its substrate proteins. Similarly, Omp85 may recognize internal sites in other OMPs lacking the C-terminal signature sequence [22], such as secretins, which are involved in type II and type III protein secretion and type IV pilus biogenesis and filamentous phage assembly; ushers, which are involved in type I pilus biogenesis; TolC and its homologs, which are involved in type I protein secretion and drugs extrusion; and Imp, which is required for the transport of lipopolysaccharide to the bacterial cell surface [40]. Alternatively, binding of such OMPs to Omp85 may be indirect and require accessory factors, such as the lipoproteins recently identified in the Omp85 complex [12], or specific chaperones, such as the pilotins in the case of the secretins [41]. The structure of Omp85 was predicted to consist of a 12-stranded β-barrel embedded in the OM with a long N-terminal periplasmic extension [8]. We propose that the periplasmic domain of Omp85 acts as a gatekeeper and recognizes nascent OMPs in the periplasm primarily via their C-terminal signature sequence. This periplasmic Omp85 domain contains several so-called POTRA (polypeptide-transport-associated) domains, which have been proposed to possess chaperone-like properties [42]. Binding of an OMP triggers a conformational change in the C-terminal domain, which is reflected in the activation of channel activity in vitro. This conformational change allows for the insertion of the OMP via the transmembrane part of Omp85, presumably in between the subunits of the Omp85 complex, which will dissociate to release the inserted OMP into the membrane, where trimerization, in the case of porins, may take place. Whereas no chaperone is required for targeting of nascent OMPs to Omp85, it seems that the insertion or folding step requires (an) additional factor(s), since no insertion of PhoE or LamB pores was monitored in planar lipid bilayers experiments. The recently identified lipoproteins that are part of the Omp85 multi-subunit complex [12] may play a role in these steps.

## Materials and Methods

### Bacterial strains and growth conditions.

E. coli K-12 strains BL21(DE3) and BL21 Star (DE3) (Novagen, Madison, Wisconsin, United States), AM1095 [43], Top10F′ (Invitrogen, Carlsbad, California, United States), and DH5α were grown at 37 °C in Luria-Bertani (LB) medium. Antibiotics were added at the following concentrations: ampicillin, 50 μg/ml; spectinomycin, 100 μg/ml. HB-1 is a capsule-deficient mutant of N. meningitidis H44/76 [44]. The N. meningitidis strain was grown in candle jars at 37 °C on GC agar (BD Biosciences, San Jose, California, United States) plates containing 2% Vitox (Oxoid, Basingstoke, United Kingdom). Liquid cultures were grown in tryptic soy broth in plastic flasks at 37 °C with aeration.

### Plasmid constructions.

The primers used to construct the plasmids are listed in Table 2. Genomic DNA of E. coli K-12 strain AM1095 was prepared by boiling a few colonies in 50 μl of H_2_O for 5 min. The lysate was centrifuged for 5 min at 13,000*g*, and the supernatant was used as template DNA for PCR. The *omp85* gene was amplified with the primer pair OyaeTfow and OyaeTrev and cloned into pCRII-TOPO (Invitrogen), yielding pCRIIyaeT. This plasmid was subsequently digested with XbaI and BamHI, and the *omp85* gene was ligated into pCL1920 [45], yielding pCLyaeT. A fragment of the *omp85* gene encoding the mature domain of the protein, i.e., without the signal peptide, was made similarly by using the primer pair OyaeTΔss and OyaeTlow in the PCR, and subcloning the fragment obtained into pCRII-TOPO. The resulting plasmid was digested with NdeI and BamHI, and the relevant fragment was ligated into pET11a (Novagen), yielding pET11ΔssyaeT.

**Table 2:**
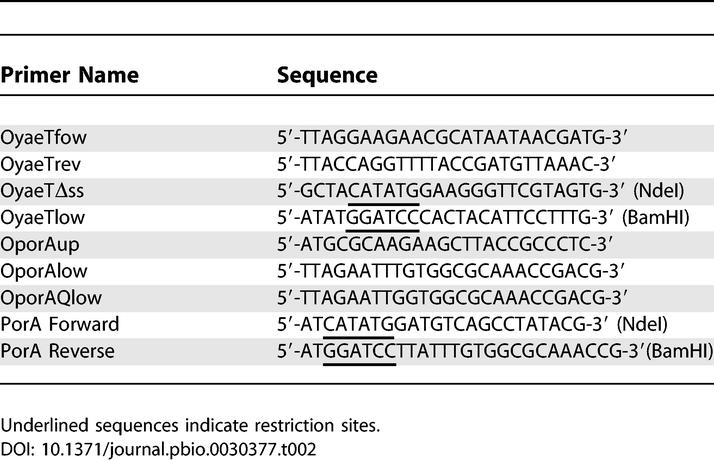
Primers Used in This Study

DNA fragments encoding wild-type *porA,* and *porA-Q* were amplified by PCR using chromosomal DNA of N. meningitidis strain HB-1 as template, OPorAup as forward primer, and either OporAlow or OporAQlow, respectively, as reverse primer (Table 2). The DNA fragments obtained were cloned into pCRII-TOPO, yielding pII-*porAwt* and pII-*porA-Q* in the correct orientation relative to the *lac* promoter, and sequences were verified. A DNA fragment encoding PorA without the signal sequence and the C-terminal phenylalanine (PorAΔF) was amplified using primers PorA Forward and PorA Reverse (Table 2), and cloned into pCR2.1-TOPO, yielding pCR2.1-PorAΔssΔF. This plasmid was subsequently digested with NdeI and BamHI, and the relevant fragment was ligated into pET11a (Novagen), yielding pET11a-PorAΔssΔF.

### Expression of recombinant Omp85 and immunization.

Strain BL21 Star (DE3) containing pET11ΔssyaeT was grown at 37 °C in 3 l LB [46] containing 50-μg/ml ampicillin. At an OD_600_ of 0.7, IPTG (0.5 mM) was added to induce expression of the recombinant gene. After another 3-h incubation at 37 °C, cells were harvested, washed with 10 mM Tris-HCl (pH 8.0), and disrupted by sonication (3 × 5 min at level 8, 40% output, Branson sonifier 450; Branson Ultrasonics Corporation, Danbury, Connecticut, United States). Omp85 was present in inclusion bodies, which were collected by centrifugation (10 min, 2,000*g,* 4 °C) and solubilized in 8 M urea containing 100 mM glycine and 20 mM Tris-HCl (pH 8.0). Residual insoluble material and membranes were removed by ultracentrifugation (100,000*g,* 1 h, 4 °C). The protein concentration in the supernatant was approximately 700 μg/ml as determined with the Pierce protein assay (Pierce Biotechnology, Rockford, Illinois, United States) using BSA as a standard. This material was used to immunize rabbits (Eurogentec, Seraing, Belgium).

### Refolding and purification of Omp85.

The folding of Omp85 was initiated by 10-fold dilution of the urea-dissolved inclusion bodies in 20 mM Tris-HCl (pH 8.0), 0.5% SB12 followed by incubation overnight at 4 °C and for 2 d at 30 °C. Small aggregates were removed by centrifugation (30 min, 8,000*g,* 4 °C). The folded protein was purified by anion-exchange chromatography on a Q-Sepharose column (Pharmacia, Uppsala, Sweden), which had been equilibrated with buffer A (20 mM Tris-HCl [pH 8.5], 0.06% *n*-decylpentaoxyethylene). The protein was eluted with a linear gradient of 0–500 mM NaCl in the same buffer. The protein concentration was determined by absorbance measurements (A_280_ of 0.1% Omp85 = 1,483).

### Size exclusion chromatography.

Fractions obtained from the Q-Sepharose column containing the folded form of Omp85 were pooled, concentrated 20-fold on a centricon YM-50 (cut-off 50 KDa) (Amicon, Millipore, Billerica, Massachusetts, United States), and dialyzed overnight against buffer A supplemented with 0.15 M NaCl (buffer C). A 200-μl sample of the resulting Omp85 solution was passed through a Superdex 200 column (Pharmacia) at a fixed flow rate of 0.4 ml/min. The void volume of the column was calculated from the elution of Blue dextran 2000 (Amersham Bioscience, Little Chalfont, United Kingdom). Elution of the standard proteins and Omp85 through the column was monitored by UV light absorption at 280 nm (Uvicord; Amersham Bioscience). Fractions of 1 ml were collected (RediFrac collector; Amersham Bioscience), and Omp85 was detected by Western blotting with an antiserum directed to Omp85.

### Cell envelope isolation.

After 3 h of growth in the presence or absence of 0.5 mM IPTG, E. coli cells were collected by centrifugation and washed with 20 mM Tris-HCl (pH 8.0) containing the protease-inhibitor cocktail “Complete” (Roche, Basel, Switzerland). Cells were broken by ultrasonic disintegration, unbroken cells were removed by centrifugation (15 min, 4,000*g,* 4 °C), and cell envelopes were collected by ultracentrifugation (40 min, 170,000*g,* 4 °C). The resulting pellet was dissolved in 5 mM Tris-HCl (pH 8.0). Neisserial cell envelopes were isolated as described [8].

### Liposome swelling assay.

Liposome swelling assays were performed as described by Van Gelder et al. [47]. In short, liposomes were prepared from 4 μmol 1,2-dioleoyl-*sn*-glycero-3-phosphocholine (Avanti Polar Lipids, Alabaster, Alabama, United States) and 1 μmol egg phosphatidyl-DL-glycerol (Avanti). After addition of Omp85, proteoliposomes were generated in 5 mM Tris-HCl (pH 7.6) containing 17% dextran T40 (Amersham Biosciences). The isotonic concentration was determined by diluting the proteoliposomes into different concentrations of raffinose in 5 mM Tris-HCl (pH 7.6). The test solutes (all in 5 mM Tris-HCl [pH 7.6]) for diffusion into the proteoliposomes were all from Sigma (St. Louis, Missouri, United States). Swelling of the proteoliposomes was monitored at 500 nm. At least four independent measurements were performed.

### Planar lipid bilayer measurements.

Planar lipid bilayers were produced as described previously [47]. In brief, lipid membranes were formed from 1% (w/v) L-α-lecithin (Sigma) in hexane at room temperature across an orifice (100–500-μm diameter) in a Teflon septum, separating two electrolyte (1 M KCl, 5 mM CaCl_2_, 10 mM Tris-HCl [pH 7.4])-containing chambers of 2.5 ml each. Omp85 protein (5–10 μg) was added to the aqueous subphase, and insertions were monitored after applying a transmembrane potential of 150 mV. The channel conductance of the pores was determined from the stepwise conductance increments. Voltage-ramp experiments were performed by applying an increasing potential from 0 to +200 or −200 mV over a time span of 200 s. To determine the voltage dependence of the probability of the channels being in an open state (P open), voltages were applied for 2 min to approach the equilibrium of channel gating, and only the current recordings of the last minute were used to calculate P open.

### Proteins and synthetic peptides.

Denatured OMPs PhoE, PhoEΔF330, PorA, and PorAΔF were produced in inclusion bodies, which were solubilized in 8 M urea containing 100 mM glycine, 20 mM Tris-HCl (pH 8.0) [30,48]. LamB was obtained in its native form [49] and denatured by boiling for 20 min in 2% SDS. Oligopeptides CterPhoE (DDIVAVGMTYQF), CterPhoEΔF (DDIVAVGMTYQ), CterPhoEKF (DDIVAVGMTYKF), random (DVTIGFYVMDAQ), CterPorA (AASVGLRHKF), and ECloopPhoE (AMHYNSDNASKD), corresponding to the C-terminus of PhoE, the same peptide but lacking the C-terminal Phe, the same peptide but with a Gln to Lys substitution at the penultimate position, the same residues as in the first peptide but in a randomized order, the C-terminus of PorA, and the first extracellular loop of PhoE, respectively, were synthesized by Sigma.

### Circular dichroism spectroscopy.

Circular dichroism (CD) spectra were recorded at 25 °C on a Jasco J-600 spectropolarimeter (Jasco, Tokyo, Japan). Samples containing 0.5 mg/ml of Omp85 in buffer A were analyzed using quartz cells with a path length of 0.1 cm. The bandwidth was 1 nm, and samples were taken each nanometer with an averaging time of 5 s. Three spectra were averaged and the contribution of the buffer subtracted.

### SDS-PAGE, Blue Native PAGE, and Western blotting.

Cell envelopes or purified protein was resuspended in SDS-PAGE sample buffer [50] containing 0.1% SDS and no β-mercaptoethanol, incubated for 10 min at either room temperature or 100 °C, and loaded on gels without SDS in the stacking and running gels. The gels were run at 14 mA in a temperature-controlled room at 4 °C to prevent denaturation of native Omp85 during electrophoresis.

Blue Native PAGE was performed as described [51] with some modifications: 6-aminohexanoic acid was omitted from the system, and samples were mixed in an equal volume of sample buffer (25 mM imidazole-HCl [pH 7.0], 17.4% [v/v] glycerol, 0.05% Coomassie blue G) prior to loading on a linear gradient gel (running gel, 5% to 20% [w/v] acrylamide; stacking gel, 3% [w/v] acrylamide). Native gel protein standards were obtained from Amersham Biosciences.

For Western blotting, proteins were transferred onto nitrocellulose membranes. The membranes were blocked for 60 min in phosphate-buffered saline (pH 7.6) (PBS) containing 0.6% nonfat dried milk, incubated for 1 h with primary antibodies against Omp85 or PorA-specific mAb Mn5C11G in blocking buffer, followed by 1-h incubation with horseradish peroxidase–conjugated goat anti-mouse or anti-rabbit IgG antibodies (Biosource, Invitrogen) in blocking buffer. Blots were developed with enhanced chemiluminescence (Amersham).

### Trypsin digestion.

Samples from cell envelopes or from in vitro folded Omp85 (0.5 mg/ml) were treated with 25 μg/ml of trypsin (Sigma) for 1 h at room temperature after which 1 mM phenylmethylsulfonyl fluoride was added, and the samples were analyzed by SDS-PAGE and Western blot. For N-terminal sequencing, the tryptic fragments were blotted from a gel containing 0.4 mM thioglycolic acid onto a polyvinylidene difluoride membrane (Millipore). The blot was stained with Coomassie blue G, and the major tryptic fragment was excised. The sample was subjected to five steps of Edman degradation at the Protein Sequencing Facility, Utrecht University.

### Immunofluorescence microscopy.

Bacteria were immobilized on polylysine-coated coverslips and fixed overnight with 2% formaldehyde in PBS. After blocking in PBS containing 3% bovine serum albumin (BSA), the coverslips were incubated with the PorA-specific mAb Mn5C11G diluted 1:500 in PBS containing 0.3% BSA, followed by incubation with Alexa-488–conjugated goat anti-mouse antibodies diluted 1:300 (Molecular Probes, Eugene, Oregon, United States). Labeling was assessed using a fluorescence microscope.

## Abbreviations

IM: inner membrane
mAb: monoclonal antibody
OM: outer membrane
OMP: outer membrane protein
